# Supplementary figures and images for: The role of pulmonary rehabilitation in idiopathic pulmonary fibrosis: An overview of systematic reviews
Source: PLoS One. 2023 Dec 21;18(12):e0295367. doi: 10.1371/journal.pone.0295367 (PMC10734956; doi:10.1371/journal.pone.0295367)

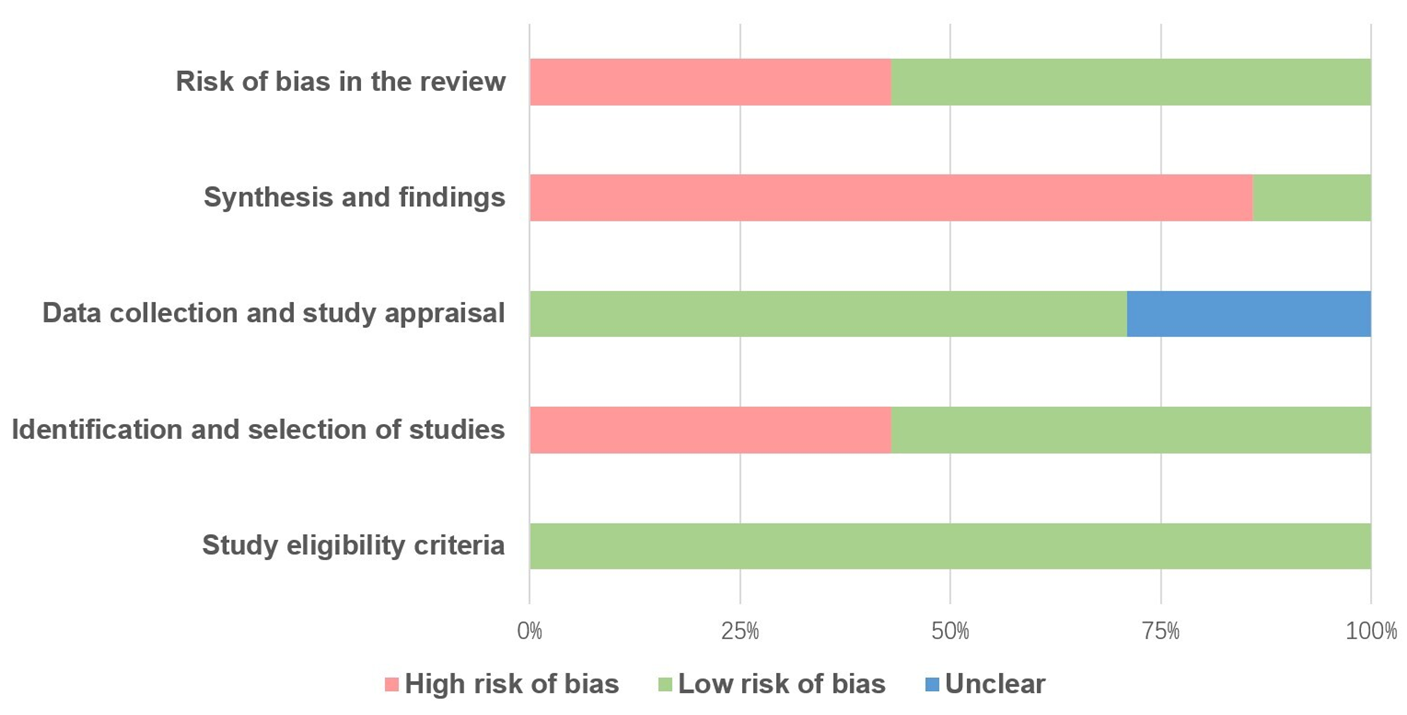

Supplement: S1 Fig — (TIF) [file pone.0295367.s002.tif]
