# Supplementary material for: The role of pulmonary rehabilitation in idiopathic pulmonary fibrosis: An overview of systematic reviews
Source: PLoS One. 2023 Dec 21;18(12):e0295367. doi: 10.1371/journal.pone.0295367 (PMC10734956; doi:10.1371/journal.pone.0295367)
Supplement: S1 Table — (DOCX) [file pone.0295367.s003.docx]

**S1 Table.Details of the literature search strategy.**

| **PubMed** |
| --- |
| 1# mh(Idiopathic Pulmonary Fibrosis or Lung Disease, Interstitial)  2# tiab (Idiopathic Pulmonary Fibrosis or Lung Disease, Interstitial or Idiopathic Pulmonary Fibroses or Pulmonary Fibroses, Idiopathic or IPF or Pulmonary Fibrosis or interstitial lung disease)  3# 1 or 2  4# mh (pulmonary rehabilitation or rehabilitation)  5# tiab (pulmonary rehabilitation or rehabilitation or rehabilitation therapy or rehabilitation training or rehabilitation program)  6# 4 or 5  7# mh (exercise therapy or muscle stretching exercises)  8# tiab (exercise therapy or muscle stretching exercises or stretching exercises or exercise training or physical training or Endurance Training or resistance training or Strength Training or upper limb training or interval training or Flexibility Training or Neuromuscular Electrical Stimulation)  9# 7or 8  10# mh (respiratory muscle training or breathing exercise)  11# tiab(respiratory muscle training or breathing exercise or inspiratory muscle training or ventilatory muscle training or respiratory exercise or respiratory training)  12# 6 or 9 or 10 or 11  13# mh meta-analysis  14# pt (meta-analysis or systematic review)  15# tiab (meta analy* or systematic review*)  16# 13 or 14 or 15  17# 3 and 12 and 16 |
| **Embase** |
| 1# exp (“fibrosing alveolitis” or lung fibrosis or interstitial lung disease)  2# (fibrosing alveolitis or Idiopathic Pulmonary Fibrosis or Idiopathic Pulmonary Fibroses or Pulmonary Fibroses, Idiopathic or IPF or Pulmonary Fibrosis or interstitial lung disease) ti.ab.kw  3# 1 or 2  4# exp (pulmonary rehabilitation or rehabilitation)  5# (pulmonary rehabilitation or rehabilitation therapy or rehabilitation training or rehabilitation program) ti.ab.kw  6# 4 or 5  7# exp (kinesiotherapy or interval training or stretching exercise or resistance training)  8# (kinesiotherapy or interval training or stretching exercise or resistance training or exercise therapy or muscle stretching exercises or exercise training or physical training Endurance Training or resistance training or Strength Training or upper limb training or interval training or Flexibility Training or Neuromuscular Electrical Stimulation)  9# 7 or 8  10#exp (respiratory muscle training or breathing exercise)  11# (respiratory muscle training or breathing exercise or inspiratory muscle training or ventilatory muscle training or breathing exercise or respiratory exercise or respiratory training) ti.ab.kw  12# 6 or 9 or 10 or 11  13# “meta-analysis”/exp  14# (meta-analysis or systematic review) it  15# (meta analy* or systematic review*) ti.ab.kw  16# 13or 14or 15  17# 3 and 12 and 16 |
| **Cochrane Library** |
| 1# MeSH descriptor:(Idiopathic Pulmonary Fibrosis or Pulmonary Fibrosis or Lung Diseases, Interstitial) explode all trees  2# ti, ab,kw (Idiopathic Pulmonary Fibrosis or Idiopathic Pulmonary Fibroses or Pulmonary Fibrosis or interstitial lung disease or Pulmonary Fibroses, Idiopathic or IPF or interstitial lung disease or Lung Diseases, Interstitial)  3# 1 or 2  4# ti, ab,kw(pulmonary rehabilitation or rehabilitation therapy or rehabilitation training or rehabilitation program)  5#MeSH descriptor: (exercise therapy) explode all trees  6#MeSH descriptor: (exercise)explode all trees  7#MeSH descriptor: (Resistance Training) explode all trees  8#MeSH descriptor: (muscle stretching exercises) explode all trees  9# ti, ab,kw (exercise therapy or exercise or resistance training or muscle stretching exercises or exercise training or physical training or upper limb training OR interval training)  10# 4 or 5 or 6 or 7 or 8 or 9  11# MeSH descriptor: (breathing exercise) explode all trees  12# ti,ab,kw(breathing exercise or respiratory muscle training or inspiratory muscle training or ventilatory muscle training or respiratory exercise or respiratory training)  13# 11 or 12  14# MeSH descriptor:( meta-analysis) explode all trees  15# pt (meta-analysis or systematic review)  16# ti,ab,kw (meta analy* or systematic review*)  17# 14 or 15 or 16  18# 3 and 10 and 17 |
| **Web of Science core collection** |
| Topic=( Idiopathic Pulmonary Fibrosis OR Lung Disease, Interstitial OR Idiopathic Pulmonary Fibroses OR Pulmonary Fibroses, Idiopathic OR IPF or Pulmonary Fibrosis OR interstitial lung disease) AND Topic=( pulmonary rehabilitation or rehabilitation or rehabilitation therapy or rehabilitation training or rehabilitation program or exercise therapy or muscle stretching exercises or stretching exercises or exercise training or physical training or Endurance Training or resistance training or Strength Training or upper limb training or interval training or Flexibility Training or Neuromuscular Electrical Stimulation or respiratory muscle training or breathing exercise or inspiratory muscle training or ventilatory muscle training or respiratory exercise or respiratory training) AND Topic=(meta analy* or systematic review* or meta-analysis or systematic review) |
