# Supplementary material for: The role of pulmonary rehabilitation in idiopathic pulmonary fibrosis: An overview of systematic reviews
Source: PLoS One. 2023 Dec 21;18(12):e0295367. doi: 10.1371/journal.pone.0295367 (PMC10734956; doi:10.1371/journal.pone.0295367)
Supplement: S2 Table — ☺, Low; ☹, High;?, Unclear. (DOCX) [file pone.0295367.s004.docx]

**S2 Table. Risk of bias of Included SRs**

| Review | Phrase 2 | | | | Phrase 3  Risk of Bias in the Review |
| --- | --- | --- | --- | --- | --- |
|  | 1.Study Eligibility  Criteria | 2. Identification and Selection of Studies | 3.Data Collection and Study Appraisal | 4. Synthesis and Finding |  |
| Guo 2023^24^  Fu 2021^25^  Cheng 2019^26^  Cheng 2018^27^  Yu 2019^28^  Mansueto 2018^29^  Lei 2022^30^ | ☺  ☺  ☺  ☺  ☺  ☺  ☺ | ☹  ☹  ☹  ☺  ☺  ☺  ☺ | ?  ☺  ?  ☺  ☺  ☺  ☺ | ☹  ☹  ☹  ☹  ☹  ☹  ☺ | ☹  ☹  ☹  ☺  ☺  ☺  ☺ |

Notes: ☺, Low; ☹, High; ?, Unclear.
