# Supplementary material for: The role of pulmonary rehabilitation in idiopathic pulmonary fibrosis: An overview of systematic reviews
Source: PLoS One. 2023 Dec 21;18(12):e0295367. doi: 10.1371/journal.pone.0295367 (PMC10734956; doi:10.1371/journal.pone.0295367)
Supplement: S3 Table — 1, Guo et al,2023 [24]; 2, Fu et al,2021 [25]; 3, Cheng et al,2019 [26]; 4, Cheng et al,2018 [27]; 5, Yu et al,2019 [28]; 6, Mansueto et al,2018 [29]; 7, Lei et al,2022 [30]. (DOCX) [file pone.0295367.s005.docx]

| **S3 Table. Report quality of included SRs/MAs.** | | | | | | | | | |
| --- | --- | --- | --- | --- | --- | --- | --- | --- | --- |
| Section | Review Items | 1 | 2 | 3 | 4 | 5 | 6 | 7 | Proportion |
|  |  |  |  |  |  |  |  |  |  |
| TITTLE | 1 | 1 | 1 | 1 | 1 | 1 | 1 | 1 | 100 |
| ABSTRACT | 2 | 0.5 | 0.5 | 0.5 | 0.5 | 1 | 0.5 | 1 | 28.57 |
|  |  |  |  |  |  |  |  |  |  |
| INTRODUCTION | 3 | 1 | 1 | 1 | 1 | 1 | 1 | 1 | 100 |
|  |  |  |  |  |  |  |  |  |  |
|  | 4 | 1 | 1 | 1 | 1 | 1 | 1 | 1 | 100 |
| METHODS | 5 | 0.5 | 0.5 | 0.5 | 0.5 | 0.5 | 0.5 | 0.5 | 0 |
|  |  |  |  |  |  |  |  |  |  |
|  | 6 | 1 | 1 | 1 | 1 | 1 | 1 | 1 | 100 |
|  | 7 | 0.5 | 0.5 | 0.5 | 1 | 0.5 | 0.5 | 0.5 | 14.29 |
|  | 8 | 0.5 | 1 | 1 | 1 | 1 | 1 | 0.5 | 71.43 |
|  | 9 | 0 | 0.5 | 0.5 | 0.5 | 1 | 1 | 1 | 42.86 |
|  | 10 | 1 | 1 | 1 | 1 | 1 | 1 | 1 | 100 |
|  | 11 | 0.5 | 0.5 | 0.5 | 1 | 1 | 1 | 1 | 57.14 |
|  | 12 | 1 | 1 | 1 | 1 | 1 | 1 | 1 | 100 |
|  | 13 | 0.5 | 1 | 1 | 1 | 1 | 1 | 1 | 85.71 |
|  | 14 | 0 | 0 | 0 | 0 | 0 | 0 | 1 | 14.29 |
|  | 15 | 0 | 0 | 0 | 0 | 0 | 0 | 1 | 14.29 |
|  | 16 | 1 | 0.5 | 1 | 1 | 1 | 0.5 | 1 | 71.43 |
| RESULTS | 17 | 1 | 1 | 1 | 1 | 1 | 1 | 1 | 100 |
|  |  |  |  |  |  |  |  |  |  |
|  | 18 | 1 | 0.5 | 1 | 1 | 1 | 1 | 1 | 85.71 |
|  | 19 | 1 | 1 | 1 | 1 | 1 | 1 | 1 | 100 |
|  | 20 | 0.5 | 0.5 | 0.5 | 0.5 | 1 | 0.5 | 0.5 | 14.29 |
|  | 21 | 0 | 0 | 0 | 0 | 0 | 0 | 1 | 14.29 |
|  | 22 | 0 | 0 | 0 | 0 | 0 | 0 | 1 | 14.29 |
|  | 23 | 0.5 | 1 | 0.5 | 0.5 | 1 | 0.5 | 1 | 42.86 |
| DISCUSSION | 24 | 0 | 0 | 0 | 0 | 1 | 0 | 1 | 28.57 |
|  |  |  |  |  |  |  |  |  |  |
|  | 25 | 0.5 | 0.5 | 0.5 | 1 | 1 | 0 | 1 | 42.86 |
|  | 26 | 0 | 0 | 0 | 1 | 1 | 0 | 1 | 42.86 |
| FUNDING | 27 | 0 | 0 | 0 | 0 | 0 | 0 | 0 | 0 |
|  | | | | | | | | | |
|  |  |  |  |  |  |  |  |  |  |
|  |  |  |  |  |  |  |  |  |  |
|  |  |  |  |  |  |  |  |  |  |
