# Supplementary material for: The role of pulmonary rehabilitation in idiopathic pulmonary fibrosis: An overview of systematic reviews
Source: PLoS One. 2023 Dec 21;18(12):e0295367. doi: 10.1371/journal.pone.0295367 (PMC10734956; doi:10.1371/journal.pone.0295367)
Supplement: Attachment — Submitted filename: Response to Reviewers.docx [file pone.0295367.s007.docx]

Dear Felix Bongomin,

Thank you so much for your letter and the comments on our manuscript entitled "The role of pulmonary rehabilitation in idiopathic pulmonary fibrosis: an overview of systematic reviews" (ID: PONE-D-23-30793R1). Thank you for your dedication and valuable suggestions on our revised manuscript. We all agree that these comments are essential to strengthen the rigor and readability of our article. Therefore, we have extensively revised the abstract of our article based on your suggestions. Revised sections are highlighted in red in the manuscript. The main corrections in the manuscript are as follows.

Responses to **Editor Comments:**

1. **Additional Editor Comments:** The results section of the abstract doesn't summarise the goal of these umbrella review. ……Kindly extensively revise the abstract.

**Response:** We have thought deeply about your comments, and we all agree that it is a highly valuable and helpful comment. We apologize for the lack of clarity in the abstract, which may prevent the editor and readers from quickly understanding the main objective of our article through the abstract. Thus, our modifications of the abstract are the following.

**Revised portion:**

(1) **Background:** The role of pulmonary rehabilitation (PR) in idiopathic pulmonary fibrosis (IPF) has been studied in several systematic reviews (SRs), but no definitive conclusions have been drawn due to the wide variation in the quality and outcomes of the studies. And there are no studies to assess the quality of relevant published SRs. This overview aims to determine the effectiveness of PR in patients with IPF and to summarize and critically evaluate the risk of bias, methodological, and evidence quality of SRs on this related topic. (The revision is on page 2, lines 24-30 of the manuscript.)

(2) **Results:** Seven SRs from 2018-2023 (including 1836 participants) on PR for the treatment of IPF were selected, all of which included patients with a definitive diagnosis of IPF. After strict evaluation by the ROBIS tool and AMSTAR-2 tool, 42.86% of the SRs had a high risk of bias and 85.71% of the SRs had critically low methodological quality in this overview. PR might be effective for patients with IPF on exercise capacity, quality of life, and pulmonary function-related outcomes, but we did not find high quality evidence to confirm the effectiveness. (The revision is on page 2, lines 38-45 of the manuscript.)

(3) We reformatted the references according to the journal's requirements but did not add or remove any references.

We appreciate your efforts again and hope that the revisions we made will be in line with PLOS ONE’s publication criteria.

Looking forward to hearing from you soon.

Sincerely,

Jiansheng Li
